# Energy Transfer from Pr^3+^ to Gd^3+^ and Upconversion Photoluminescence Properties of Y_7_O_6_F_9_:Pr^3+^, Gd^3+^

**DOI:** 10.3390/ma15217680

**Published:** 2022-11-01

**Authors:** Yang Sun, Yangbo Wang, Chengchao Hu, Xufeng Zhou, Jigong Hao, Wei Li, Huaiyong Li

**Affiliations:** School of Materials Science and Engineering, Liaocheng University, Liaocheng 252059, China

**Keywords:** upconversion, Y_7_O_6_F_9_, phosphors, energy transfer

## Abstract

Upconversion materials have numerous potential applications in light energy utilization due to their unique optical properties. The use of visible light excitation to obtain ultraviolet emission is a promising technology with broad application prospects, while relevant research is absent. A series of Pr^3+^, Gd^3+^ doped Y_7_O_6_F_9_ phosphors were synthesized by traditional solid–state reaction. X-ray diffraction, scanning electronic microscopy, steady-state photoluminescence spectra, a decay dynamic, and upconversion emission spectra of the samples were studied. Under the excitation of 238 nm, the energy transfer from Pr^3+^ to Gd^3+^ was realized and a strong ultraviolet B emission due to the ^6^P_7/2_→^8^S_7/2_ transition of the Gd^3+^ ions was achieved. Under the excitation of a 450 nm blue laser, Pr^3+^ absorbed two blue photons to realize the upconversion process and then transferred the energy to Gd^3+^ to obtain the ultraviolet B emission.

## 1. Introduction

Upconversion (UC) luminescence refers to the nonlinear process of emitting a high-energy photon when two or more low-energy photons are absorbed. Because of its unique physical and chemical properties, UC luminescence has attracted extensive attention over the past few years. Most of the research on UC is based on the excitation in the infrared region to obtain the emission in the visible region and has made enormous progress, which is widely applied in solar cells, biological imaging, oxygenic photosynthesis, solid–state lasers, fluorescent probes, and photodynamic therapy [1,2,3,4,5]. Taking into account factors, such as conversion efficiency, research on the use of abundant visible energy to realize visible-to-ultraviolet (UV) conversion is relatively lacking. UV radiation refers to electromagnetic waves with wavelengths ranging from 100 nm to 400 nm, which is very significant in physics, medicine, the environment, and other aspects. UV light can be divided into ultraviolet A (UVA), ultraviolet B (UVB), and ultraviolet C (UVC), and their wavelength ranges are 400–315 nm, 315–280 nm, and 280–100 nm, respectively. It is necessary and meaningful to seek a method and mechanism to obtain UV emission. Many researchers have made great efforts to explore UV lasers, using UC luminescent materials and nonlinear crystal technology to obtain deep UV lasers [6,7,8]. At present, using UVB fluorescent lamps is a common method to treat skin diseases, such as psoriasis, vitiligo, and skin burn. Various studies have shown that the most effective range is in the long wave part of the UVB spectrum, that is, between 305 nm and 315 nm. In addition to phototherapy, UVB also has potential advantages in other areas. Because UVB has the visible–blind feature, UV cameras can detect UVB radiation in indoor-lighting environments and can effectively control the interference of solar light sources. Therefore, it has potential application in the field of optical tagging [9,10,11,12]. The use of low-energy light as the excitation light source to obtain high-energy UV emission and broaden the application range of the UV region has important research significance. 

For some rare earth ions (RE^3+^), such as Pr^3+^, Gd^3+^, Tb^3+^, and Ce^3+^, the emission corresponding to the transition between some energy levels is located in the UV band so it can be used as the UV luminous activator. The emission of Pr^3+^ extends from UV to infrared radiation due to its interaction between abundant 4f energy levels and the 4f5d state. In various phosphors doped with Pr^3+^ ions, the transition of ^3^H_4_ to ^3^P_J_ or ^1^D_2_ and then to the 4f5d state has been realized under visible excitation, and the UV region emission of the 4f5d state has been obtained [6,13,14,15,16,17]. The luminescence properties of Pr^3+^ ions largely depend on the host lattice. An important criterion for realizing the visible-to-UV mechanism is that the 4f5d energy level of Pr^3+^ ions is lower than the ^1^S_0_ energy level, and if the host lattice crystal field causes a band energy that is too high, the second excitation step cannot be achieved [14,16,17]. The Gd^3+^ ion shows an ff transition and a characteristic emission at about 313 nm. Furthermore, it has no absorption in visible light due to its unique energy-level structure. Therefore, reports on Gd^3+^ ions’ luminescence are mainly ordinary photoluminescence (PL) under high-energy UV excitation. The 4f5d interconfigurational transition of Pr^3+^ overlaps with the ^8^S_7/2_→^6^D_J_ transition of Gd^3+^, which can result in an efficient energy transfer. Thus, the UVB emission of the Gd^3+^ ions excited by visible light can be realized [12,18,19,20]. The quantum efficiency is determined by the nonradiative process of the host lattice. High phonon vibration will increase the nonradiative relaxation rate and, thus, reduce the quantum efficiency [21]. In contrast, fluoride generally has the advantages of low phonon energy (less than 350 cm^−1^) and a wide transparent region, but it is less chemically stable, easily oxidized, susceptible to moisture, and difficult to prepare and handle. Oxide has good stability, but its phonon energy is relatively high. Oxyfluorides combine the advantages of fluoride and oxide, have moderate phonon cutoff energy and good chemical stability, and are generally considered an ideal host [22,23,24,25]. The Y_7_O_6_F_9_ crystal has high photochemical stability and a suitable crystal environment, which has great potential to be used as UV UC laser materials. 

In this work, we have synthesized Y_7_O_6_F_9_:Pr^3+^, Gd^3+^ phosphors via the solid–state synthesis method and investigated their optical properties. The energy transfer from Pr^3+^ to Gd^3+^ was analyzed. The UC emission from Gd^3+^ in the UV region upon the excitation of blue light was investigated.

## 2. Experimental

### 2.1. Synthesis

Y_7_O_6_F_9_:Pr^3+^ and Y_7_O_6_F_9_:Pr^3+^, Gd^3+^ phosphors were synthesized by a traditional solid–state reaction. Stoichiometric amounts of Y_2_O_3_, Pr_6_O_11_, and Gd_2_O_3_ and excessive NH_4_F were mixed and ground (the ratio of the rare earth elements to fluorine was 1:2). For the Pr^3+^ and Gd^3+^ doping, a stoichiometric amount of Y^3+^ was omitted. The mixtures were then sintered at 1120 ℃ for 30 min in a tube furnace. The samples were cooled down naturally and crushed for further characterization. 

### 2.2. Characterization

The crystal structure of the synthesized samples was identified using powder X-ray diffraction (XRD) analysis by using a Bruker D8 Advance diffractometer and Cu Kα radiation as the incident radiation. The patterns were collected within a 2θ range of 5°~90°. The morphology of the powder was characterized on a Zeiss Merlin Field Emission Scanning Electron Microscope (FE-SEM). Energy-dispersive X-ray spectroscopy (EDS) was performed on the accessories to the FE-SEM. Steady-state emission and excitation spectra were measured on a Hitachi F-7000 fluorescence spectrophotometer using a 150 W xenon lamp as the excitation source. Fluorescence decays were collected on an Edinburgh Instruments FS5 fluorescence spectrometer with a μs flash lamp as the excitation source. UC emission spectra were obtained by exciting the sample with a 450 nm continuous wave (CW) laser with a maximum output power of 2 W (Changchun New Industries Optoelectronics Technology, Changchun, China).

## 3. Results and Discussion

### 3.1. Phase and Structure Analysis

**Figure 1:**
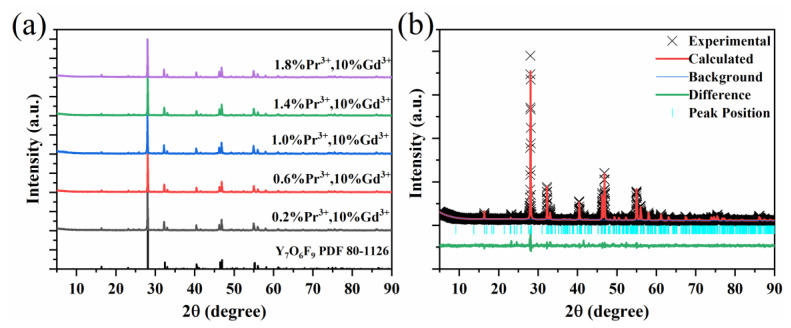
(**a**) XRD patterns of Y_7_O_6_F_9_:Pr^3+^, Gd^3+^ phosphors. (**b**) Results of the Rietveld refinement on Y_7_O_6_F_9_:1.4%Pr^3+^, 10%Gd^3+^.

The XRD patterns of the Y_7_O_6_F_9_:Pr^3+^, Gd^3+^ powders are presented in Figure 1a. The diffraction patterns of all the samples agree with the standard card of Y_7_O_6_F_9_ (JCPDS No. 801126), and no detectable impurity peaks corresponding to the second phase are observed. The Y_7_O_6_F_9_ crystal structure is highly tolerable, and a solid solution is formed, despite the great difference in the ionic radius of Y^3+^ and Gd^3+^. A Rietveld analysis was performed on Y_7_O_6_F_9_:1.4%Pr^3+^, 10%Gd^3+^ by using the GSAS program with the structure parameters as the initial input. The calculated and experimental results, as well as their differences of the Y_7_O_6_F_9_:1.4%Pr^3+^, 10%Gd^3+^ sample, are shown in Figure 1b. The obtained cell parameters of Y_7_O_6_F_9_:1.4%Pr^3+^, 10%Gd^3+^, as well as the reliability, are listed in Table 1. The reliability parameters are R_wp_ = 8.42%, R_p_ = 6.39%, χ^2^ = 2.276, which supports the conclusion that the prepared samples are pure phase.

**Figure 2:**
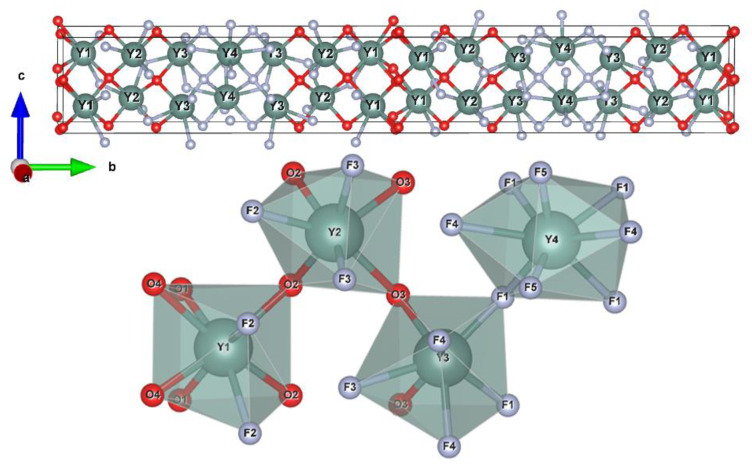
Crystal structure of Y_7_O_6_F_9_ (above) and four kinds of coordination for Y^3+^ (below).

The crystal structure of Y_7_O_6_F_9_ was visualized by using Vesta software and is shown in Figure 2. There are four different kinds of Y^3+^ sites, five F^−^ sites, and four O^2−^ sites in the lattice. The coordination environments of Y^3+^ are also shown in Figure 2. The Y(1) atom is surrounded by six O^2−^ and two F^−^ atoms in an 8d position, Y(2) by four O^2–^ and three F^−^ in an 8d position, Y(3) by two O^2−^ and five F^−^ in an 8d position, and Y(4) is surrounded by eight F^−^ atoms in a 4c Wyckoff position. 

### 3.2. SEM Image of the Y_7_O_6_F_9_:Pr^3+^, Gd^3+^ Phosphor

**Figure 3:**
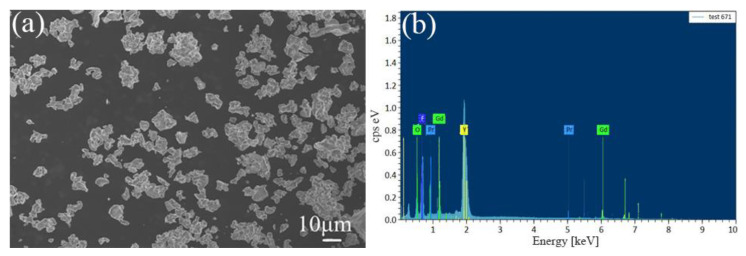
(**a**) SEM and (**b**) EDS images of Y_7_O_6_F_9_:1.4%Pr^3+^, 10%Gd^3+^ phosphor.

In order to examine the surface morphology and average crystal size, an SEM image of Y_7_O_6_F_9_:1.4%Pr^3+^, 10%Gd^3+^ was checked and is shown in Figure 3a. The powder is composed of irregular particles, with an average diameter of about 6.3 µm. The EDS analysis in Figure 3b confirms the existence of Y, O, F, Pr, and Gd elements in the material.

### 3.3. PL Properties of the Y_7_O_6_F_9_:Pr^3+^ Phosphor 

**Figure 4:**
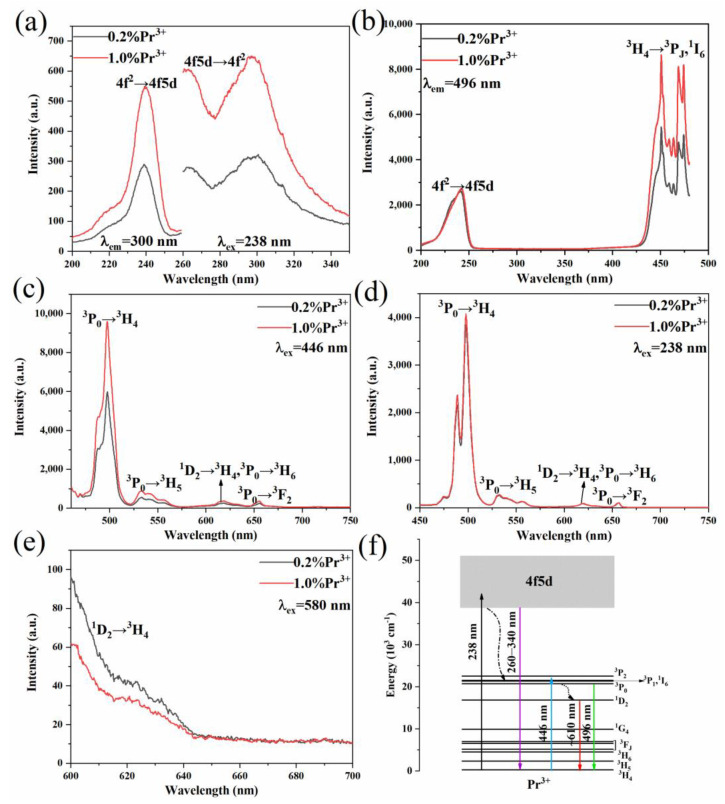
Basic PL properties of Y_7_O_6_F_9_:xPr^3+^ (x = 0.2%, 1.0%) phosphors. (**a**) Excitation (λ_em_ = 300 nm) and emission (λ_ex_ = 238 nm) spectra. (**b**) Excitation spectra (λ_em_ = 496 nm). (**c**) Emission spectra (λ_ex_ = 446 nm). (**d**) Emission spectra (λ_ex_ = 238 nm). (**e**) Emission spectra (λ_ex_ = 580 nm). (**f**) Energy level diagram of Pr^3+^. Solid lines represent absorption and emission, and dashed black lines represent nonradiative relaxation.

To investigate the interaction between the Pr^3+^ ions in the Y_7_O_6_F_9_ matrix, the excitation and emission spectra of Pr^3+^ in the UV and visible regions were studied. Figure 4a shows the excitation and emission spectra of Pr^3+^ in the UV region. The excitation spectrum was obtained by monitoring the emission at 300 nm. There is an excitation band within the range of 200–250 nm with a maximum of 238 nm, which can be assigned to the 4f^2^→4f5d transition of Pr^3+^. The emission spectra consist of two emission bands with the maxima at 262 nm and 300 nm upon excitation of UV light at 238 nm, which can be attributed to the 4f5d→4f^2^ interconfigurational transition of Pr^3+^. It can also be observed that the intensity enhanced with the Pr^3+^ concentration, increasing from 0.2 mol% to 1.0 mol%. The excitation spectra shown in Figure 4b are of the emission at 496 nm, which contain two different types of transitions. The narrow line excitation peaks in the visible region are attributed to the ^3^H_4_→^3^P_0, 1, 2_, ^1^I_6_ transitions of Pr^3+^. The broad excitation bands centered at 238 nm are due to the transition of 4f^2^→4f5d. The presence of this band suggests the nonradiative relaxation from the 4f5d state to the 4f^2^ state. The 4f states stoke the emission generated by the 446 nm excitation is measured, as shown in Figure 4c. The strongest peak at 496 nm originated from the transition of ^3^P_0_→^3^H_4_, indicating that the ^3^P_0_ level is 20,161 cm^−1^ higher than the ^3^H_4_ level. Upon the excitation of blue light, ^3^P_J_ (J = 0, 1, 2), or the ^1^I_6_ states, could be populated, while a rapid relaxation generally happens to the ^3^P_0_ state. One can notice that the spectra are dominated by the emission due to ^3^P_0_→^3^H_J_ (J = 4, 5, 6) transition whether the Pr^3+^ doping concentration is 0.2 mol% or 1.0 mol%, and the emission from the ^1^D_2_→^3^H_4_ transition is extremely weak. Figure 4d shows the emission spectra at 450~750 nm upon excitation at 238 nm. The emission is similar to those shown in Figure 4c. It comes from the relaxation of Pr^3+^ to the lower energy level when it is excited by the 4f5d state and is then followed by the transition within the 4f^2^ configuration. Through 580 nm of light used to excite the electrons from the ground state ^3^H_4_ to the ^1^D_2_ level, the spectra show an emission band from 600 nm to 640 nm, as shown in Figure 4e. However, the intensity changes in a manner opposite to Figure 4c; the emission intensity decreases with the increase of the Pr^3+^ ion concentration, indicating that the quenching of the ^1^D_2_ state is very severe, which may be caused by two reasons. The ^1^D_2_ level can be populated through the ^3^P_0_→^1^D_2_ multi-phonon relaxation and [^3^P_0_, ^3^H_4_]→[^3^H_6_,^1^D_2_] cross-relaxation. It is known that the energy range between ^3^P_0_ and ^1^D_2_ is about 3866cm^−1^, and the phonon energy of Y_7_O_6_F_9_ is lower (about 450 cm^−1^) so that 8.5 phonons are required to achieve relaxation from ^3^P_0_ to ^1^D_2_. In order for the equivalent phonon number for the multi-phonon relaxation process to occur, it must be less than 4~5 phonons; so, the probability of occurrence is lower. The quenching of the ^1^D_2_ energy level may also occur. The [^1^D_2_, ^3^H_4_]→[^1^G_4_, ^3^F_3,4_] transitions yield a population of ^1^G_4_ and ^3^F_3,4_ levels [26,27,28]. The transition and relaxation between the relevant energy levels mentioned above under 238 nm and 446 nm blue excitations can be explained by the electron populating processes (see Figure 4f).

### 3.4. PL Properties of the Y_7_O_6_F_9_:Pr^3+^, Gd^3+^ Phosphor

**Figure 5:**
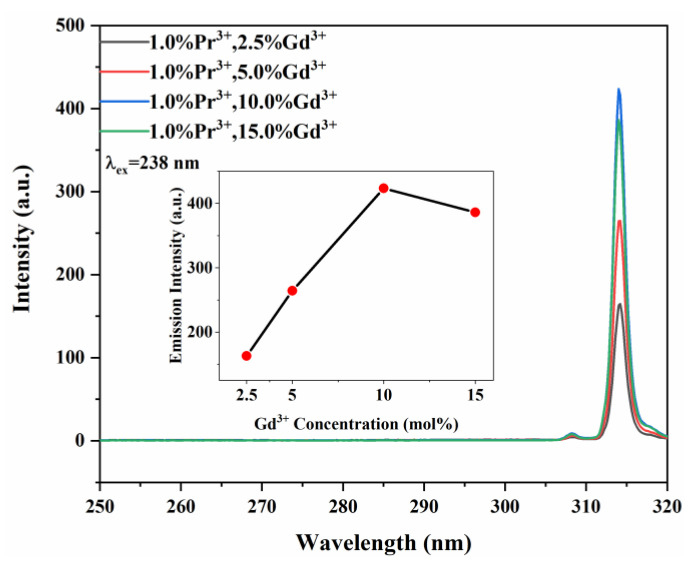
Emission spectra (λ_ex_ = 238 nm) of Y_7_O_6_F_9_:1.0%Pr^3+^, xGd^3+^ (x = 2.5%, 5%, 10%, 15%) phosphors.

Figure 5 shows the emission spectra of Y_7_O_6_F_9_: 1.0%Pr^3+^, xGd^3+^ as a function of the Gd^3+^ concentration. It shows that the intensity of the Gd^3+^ emission increases with the Gd^3+^ content till 10 mol%, and then it drops.

**Figure 6:**
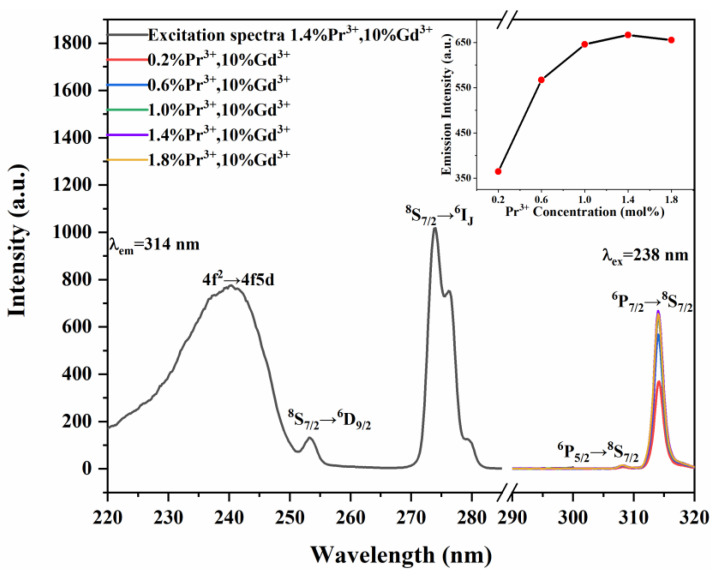
Excitation (λ_em_ = 314 nm) and emission spectra (λ_ex_ = 238 nm) of Y_7_O_6_F_9_:xPr^3+^, 10%Gd^3+^ (x = 0.2%, 0.6%, 1.0%, 1.4%, 1.8%) phosphors.

Figure 6 illustrates the excitation spectra-monitored emission wavelength at 314 nm. The excitation spectra consist of several excitation peaks of 253 nm, 274 nm, and 276 nm, corresponding to the electronic transition of Gd^3+^ and a broad absorption band from 220 to 250 nm. The broad excitation band can be attributed to the 4f^2^→4f5d transition of electrons in the Pr^3+^ ions. The excitation peaks at 253 nm, 274 nm, and 276 nm correspond to the ^8^S_7/2_→^6^D_9/2_, ^8^S_7/2_→^6^I_11/2_, ^8^S_7/2_→^6^I_9/2_ transitions of the Gd^3+^ ions, respectively. Through monitoring the emission of Gd^3+^, the broad and intense absorption peaks of Pr^3+^ were obtained, and it can be concluded that most of the excitation energy is absorbed by the Pr^3+^ ions. 

The emission spectra of the Y_7_O_6_F_9_:xPr^3+^, 10%Gd^3+^ phosphors in the UV region upon excitation at 238 nm are also shown in Figure 6. The luminescence intensity increases with the increasing Pr^3+^ doping concentration, reaching a maximum at 1.4 mol%, and then it no longer increases, indicating the onset of concentration quenching effects, which can be clearly seen from the inset. From these emission spectra, one can clearly observe the sharp and intense emission peak at 314 nm and a lower intensity peak at 309 nm, which are designated as the ^6^P_7/2_→^8^S_7/2_ and ^6^P_5/2_→^8^S_7/2_ transition of Gd^3+^, respectively. The process is that when the electrons in the Pr^3+^ ions transition to the 4f5d level by absorbing the excitation energy, their energy is transferred to the excitation level of the Gd^3+^ ions. Gd^3+^ relaxes to the ground state and produces UVB emission. It shows that the strategy of energy transfer from Pr^3+^ to Gd^3+^ is effective. Gd^3+^ ions possess an interconfiguration 4f-4f transition and have a wide band gap greater than 30,000 cm^−1^, which reduces the multi-phonon transition [29]. The absence of a broadband emission from the 4f5d→4f^2^ transition for Pr^3+^ also indicates that the energy transfer from Pr^3+^ to Gd^3+^ is efficient.

**Figure 7:**
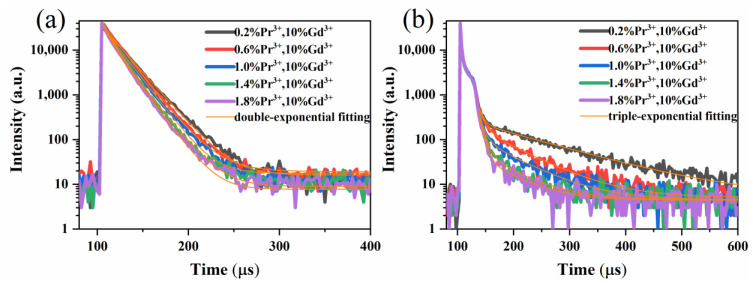
(**a**) ^3^P_0_ decay curves (λ_ex_ = 446 nm, λ_em_ = 496 nm). (**b**) ^1^D_2_ decay curves (λ_ex_ = 580 nm, λ_em_ = 602 nm) of the Y_7_O_6_F_9_:xPr^3+^, 10%Gd^3+^ (x = 0.2%, 0.6%, 1.0%, 1.4%, 1.8%) phosphors.

The fluorescence decay curve of the ^3^P_0_→^3^H_4_ transition of Y_7_O_6_F_9_:xPr^3+^, 10%Gd^3+^ was monitored under the excitation of a 446 nm pulsed xenon lamp, as shown in Figure 7a. It is observed that all the samples show quite similar decay behavior and the decay curves can be well-fitted as a double exponential function. The double exponential model is given below:

(1) $$I_{t}=A_{1}\exp\left(-t/\tau_{1}\right)+A_{2}\exp\left(-t/\tau_{2}\right)$$

where I_t_ is the emission intensity at time t, A_1_ and A_2_ are constants, and τ_1_ and τ_2_ are the partial decay lifetimes of the exponential components. The average lifetime, τ, of the ^3^P_0_ level is calculated from the following equation:

(2) $$\tau =\left(A_{1}\tau_{1}{}^{2}+A_{2}\tau_{2}{}^{2}\right)/\left(A_{1}\tau_{1}+A_{2}\tau_{2}\right)$$

The fluorescence lifetime values are given in Table 2 and decreased from 18.8 μs to 13.8 μs with an increasing Pr^3+^ concentration, indicating that the nonradiative relaxation of the Pr^3+^ ions increases. As mentioned above, the multi-phonon relaxation rate is related to the phonon energy of the matrix, so the cross-relaxation between adjacent Pr^3+^ ions may be the main reason for the fast decay of the ^3^P_0_ state. In addition, the lifetime of the ^1^D_2_ level was measured under 580 nm of pulse excitation, as shown in Figure 7b, and the curve was fitted by a more complicated triple exponential equation. The triple exponential model is:

(3) $$I_{t}=A_{1}\exp\left(-t/\tau_{1}\right)+A_{2}\exp\left(-t/\tau_{2}\right)+A_{3}\exp\left(-t/\tau_{3}\right)$$

The average lifetime, τ, of the ^1^D_2_ fluorescence was obtained applying the following expression:

(4) $$\tau =\left(A_{1}\tau_{1}{}^{2}+A_{2}\tau_{2}{}^{2}+A_{3}\tau_{3}{}^{2}\right)/\left(A_{1}\tau_{1}+A_{2}\tau_{2}+A_{3}\tau_{3}\right)$$

The lifetime values are also given in Table 2. It can be seen that the lifetime value of ^1^D_2_ is much longer than that of the ^3^P_0_ state, which is due to its own singlet spin multiplicity and spin-forbidden transition [27]. With the increase of the Pr^3+^ ion concentration, the lifetime value also shows a sharp decrease trend. It may be that the [^1^D_2_, ^3^H_4_]→[^1^G_4_, ^3^F_3,4_] cross-relaxation process between the Pr^3+^ ions leads to an increase in the ^1^D_2_ nonradiative relaxation rate, which leads to a decrease in the lifetime value. 

**Figure 8:**
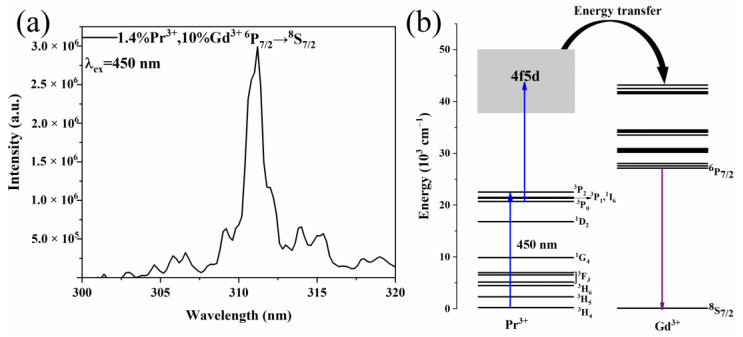
(**a**) UC Emission spectra (λ_ex_ = 450 nm) of the Y_7_O_6_F_9_:1.4%Pr^3+^, 10%Gd^3+^. The output power was 0.38 W. (**b**) UC energy transfer mechanism of Pr^3+^ to Gd^3+^.

The UC properties of the Y_7_O_6_F_9_:1.4%Pr^3+^, 10%Gd^3+^ phosphor were investigated under excitation with a CW blue laser at 450 nm, as shown in Figure 8a. It can be clearly seen that the spectra were dominated by the emission peak of Gd^3+^. Considering that there is no intermediate state between the ^8^S_7/2_ and ^6^P_7/2_ states of Gd^3+^, the excitation at 450 nm could not directly excite Gd^3+^, indicating that the energy absorbed by Gd^3+^ comes from the 4f5d energy level of Pr^3+^. Effective UVB emission of Gd^3+^ was obtained by exciting the Pr^3+^ ions, clearly demonstrating that the energy transfer from Pr^3+^ to Gd^3+^ must be very successful. The energy level diagrams of Pr^3+^ and Gd^3+^ and the UC process of the material system are plotted in Figure 8b. Under blue light excitation, the two-step excitation dominates the UC process. The first excitation brings Pr^3+^ from the ground state, ^3^H_4_, to the excited state, ^3^P_J_. A rapid relaxation happens to the ^3^P_0_ state and then Pr^3+^ is excited from the intermediate state to the higher energy 4f5d state. The Gd^3+^ ions can extract the excitation energy of Pr^3+^ to transition to the ground state.

## 4. Conclusions

In conclusion, Y_7_O_6_F_9_ doped with Pr^3+^ and Gd^3+^ phosphors were successfully synthesized by the traditional solid–state reaction method. XRD data of the Y_7_O_6_F_9_ phosphor are in good agreement with the standard JCPDS data. SEM images showed the agglomeration of irregular particles, and EDS confirmed the presence of all the elements in the phosphor. Through an efficient energy transfer from Pr^3+^ to Gd^3+^, a strong narrow-band UVB emission from the Gd^3+^ ion could be obtained under 238 nm of excitation. Under blue laser excitation at 450 nm, the UVB emitting of the Gd^3+^ ions was obtained, which originated from Pr^3+^ absorbing two blue photons to the 4f5d configuration, followed by an energy transfer to Gd^3+^. Our findings suggest that Y_7_O_6_F_9_:Pr^3+^, Gd^3+^ is a promising candidate for visible-to-UV phosphors and opens up new options for applications requiring UV radiation, such as phototherapy treatments and optical tagging.

## Figures and Tables

**Table 1:** Cell parameters of Y_7_O_6_F_9_:1.4%Pr^3+^, 10%Gd^3+^ obtained from the Rietveld refinement.

Formula	Y_7_O_6_F_9_
Crystal system	orthorhombic
Space group	Abm2(39)
Cell parameters	a = 5.4191(1) Å
	b = 38.8414(8) Å
	c = 5.5477(1) Å
	α = β = γ = 90°
	V = 1167.702 Å^3^
2θ Range	5° ≤ 2θ ≤ 90°
Reliability factors	R_wp_ = 8.42%
	R_p_ = 6.39%
	χ^2^ = 2.276

**Table 2:** The calculated lifetimes for ^3^P_0_ decay and ^1^D_2_ decay.

Samples	^3^P_0_ lifetimes in µs	^1^D_2_ lifetimes in µs
0.2%Pr^3+^, 10%Gd^3+^	18.84(2)	132.40(1)
0.6%Pr^3+^, 10%Gd^3+^	17.21(9)	58.11(2)
1.0%Pr^3+^, 10%Gd^3+^	15.89(1)	54.71(1)
1.4%Pr^3+^, 10%Gd^3+^	14.40(6)	41.10(7)
1.8%Pr^3+^, 10%Gd^3+^	13.78(2)	28.45(2)

## Data Availability

Not applicable.
